# Total Antioxidant Capacity of Serum Determined Using the Potassium Permanganate Agar Method Based on Serum Diffusion in Agar

**DOI:** 10.1155/2015/406071

**Published:** 2015-08-11

**Authors:** Ying Zhou, Meijuan Zhang, Hui Liu

**Affiliations:** College of Medical Laboratory, Dalian Medical University, Dalian 116044, China

## Abstract

*Objectives*. To develop a new method for determining total antioxidants in serum and to evaluate the total antioxidant capacity of organisms. *Design and Methods*. Sodium hyposulfite (Na_2_S_2_O_3_) and serum were used to evaluate the linearity and precision of the potassium permanganate agar method. The area of serum diffusion in samples from 30 intensive care unit (ICU) patients compared with 44 healthy subjects was determined by the potassium permanganate agar method. *Results*. The linearity (*R* ^2^ in the linear experiment of Na_2_S_2_O_3_ was 0.994; *R* ^2^ in the linear experiment of serum was 0.987) and precision (coefficient of variation of area of high level serum diffusion within-run, between-run, and between-day and coefficient of variation of area of low serum diffusion within-run, between-run, and between-day were all less than 10%) were acceptable using the potassium permanganate agar method. Total antioxidants of serum between the ICU group and the healthy group were different (*p* = 0.002, two tailed). *Conclusions*. Total antioxidants in serum can be determined by the potassium permanganate agar method. The total antioxidant capacity of an organism can be evaluated by the amount of total antioxidants in serum.

## 1. Introduction

Biological free radicals are normal products of metabolism, including reactive oxygen species (ROS) and reactive nitrogen species (RNS), and ROS is the dominant for over 95% of the biological free radicals. In normal conditions, the production and removal of free radicals are balanced as they play an important role in biological systems [1, 2]. Once this balance is disturbed, free radicals can damage cells, tissue, and organs which cause cancer [3], cardiac diseases [4], brain diseases [5], aging [6], and so on. In the circulatory system, the concentration of human serum albumin (HSA) in plasma is 35–50 g/L. HSA constitutes 60% of total plasma proteins and is important in maintaining endogenous antioxidant function in organisms [7]. Many assays have been used to determine the total antioxidant capacity (TAC) of serum, but these methods all have some limitations. The total radical trapping antioxidant parameter (TRAP) [8, 9] assay and oxygen radical absorbance capacity (ORAC) [10, 11] assay are based on hydrogen atom transfer (HAT) reactions; however, special devices are necessary and the reactions are complicated. The trolox equivalent antioxidant capacity (TEAC) [12–15] assay, 2,2-diphenyl-1-picrylhydrazyl (DPPH) [16, 17] assay, ferric reducing antioxidant power (FRAP) [18, 19] assay, and the cupric ion reducing antioxidant capacity (CUPRAC) [20, 21] assay are based on electron transfer (ET) reactions. However, the TEAC assay has poor accuracy and the results may be affected by the time chosen to read the absorbance. However, the DPPH method uses MeOH as the solvent, but MeOH will subvert the order of the actual antioxidant activity. The FRAP assay requires acetate buffer, at a pH of 3.6. Mercapto compounds (SH-) in sample interfere with the results and any compound with a suitable redox potential will also drive Fe^III^-TPTZ reduction. The CUPRAC assay can detect parts of antioxidants in serum, as the standard electrode potential of Cu^2+^/Cu^+^ is low. In addition, the buffer added to the reaction system also interferes with the results. Thus, in this study, potassium permanganate (KMnO_4_) was used as the oxidant to determine the TAC in serum [22], with the aim of providing a method with good linearity and precision for detecting total antioxidants in serum and simplifying experimental procedures.

Redox titration can detect the quantity of inorganic or organic compounds in a direct or indirect manner. KMnO_4_ is an ideal oxidant; it has strong oxidizing power and few nonredox reactions; the standard electrode potential of MnO_4_^−^/MnO_2_ is as high as 1.695 V. Mn^7+^ can be reduced completely to Mn^2+^ only in a strong acidic environment. Because studies have shown that pH can interfere with the results of redox reactions [23], the redox reaction should be set in a neutral environment close to physiological pH 7.35–7.45. However, in a neutral environment, an abundant brown precipitate of MnO_2_ is generated in the potassium permanganate titration method, and the precipitate MnO_2_ interferes with the judgement of end point. Thus, the potassium permanganate agar method is a better method for determining the TAC in serum. The potassium permanganate agar method is based on potassium permanganate redox titration but requires less reagent and sample than the potassium permanganate redox titration method. Serum diffuses in the potassium permanganate agar, and the color of the redox reaction area changes due to the decrease in Mn^7+^. A brown circle appears 24 hours later in the redox reaction area, while the nonredox reaction area becomes red. The amount of total antioxidants in serum can be determined by measuring the area of serum diffusion and the redox state of the organism can be evaluated by the amount of total antioxidants in serum.

## 2. Methods

### 2.1. Serum Specimens

Serum specimens were collected from the First Hospital Affiliated with Dalian Medical University and the Second Hospital Affiliated with Dalian Medical University. Thirty intensive care unit (ICU) patients were regarded as the experimental group and included 20 males and 10 females, with mean age of 63.83 ± 15.20 years. Forty-four healthy subjects were regarded as the healthy group and included 31 males and 13 females, with mean age of 57.80 ± 11.45 years. The diagnoses in the experimental group included stomach neoplasms, brain neoplasms, food poisoning, and so forth. Serum specimens were stored at −20°C after collection and thawed at room temperature before the experiment. The study was approved by the Ethical Committee of Dalian Medical University.

### 2.2. Methods

1.5 g agar was dissolved in 100 mL distilled water and heated for 3 minutes at 100°C. When the temperature of the agar solution was cooled down to 55°C, 15 mL 0.003 mol/L KMnO_4_ was mixed with 70 mL agar solution. 7 mL potassium permanganate agar solution was added to the dish. The diameter of the dish was 7 cm; thus, the thickness of the potassium permanganate agar was 0.18 cm. When the potassium permanganate agar solidified, an aperture was created in the center of the potassium permanganate agar; the agar aperture was 6 mm. 30 *μ*L serum was added to the aperture and covered with 6 mL liquid paraffin on surface of the potassium permanganate agar. The potassium permanganate agar was stored in dark at 4°C for 24 h. The diameter of the brown circle was measured, and the area of serum diffusion was calculated (including the diameter of the aperture) using the following formula: *S* = *π* × (*d*/2)^2^, where *S* represented the area of serum diffusion and *d* represented the diameter of the brown circle.

### 2.3. Linear Experiment

0.01 mol/L of the standard reductant, Na_2_S_2_O_3_, and pooled serum were diluted to their 100%, 80%, 60%, 40%, and 20% concentrations. A parallel sample was prepared for each concentration. The diameter of the brown circle was measured and the area of Na_2_S_2_O_3_ diffusion as well as serum diffusion was calculated.

### 2.4. Precision Experiment

Referring to the document on Evaluation of Precision Performance of Quantitative Measurement Methods, approved guideline-second edition (EP5-A2) [24], serum from 5 healthy people was pooled and we diluted the pooled serum to a concentration of 100% (regarded as high level) and 50% (regarded as low level), added them to potassium permanganate agar, measured the diameter of brown circle, calculated the area of serum diffusion, and included a parallel sample for each run, two runs every day for twenty days in total. The interval between two runs was longer than 2 hours.

### 2.5. Methodology Comparison

A serum sample was diluted with water to its 100%, 80%, 60%, 40%, and 20% concentrations. 30 *μ*L serum at each diluted level was added to the potassium permanganate agar. The diameter of the brown circle of each diluted level was measured and the area of serum diffusion was calculated. The above 5 diluted levels of serum were measured using our described method and previously reported iodine redox titration [22]. The areas of serum diffusion in the potassium permanganate agar and optical density (OD) in iodine redox titration were compared.

### 2.6. Sample Test

The serum samples from healthy subjects and the ICU patients were added to potassium permanganate agar and the diameter of the brown circle was measured and the area of serum diffusion was calculated.

### 2.7. Statistical Analysis

The statistical software package SPSS 13.0 was used to evaluate the results. The nonparametric test was used to compare the results. A value of *p* < 0.05 (two tailed) was considered significant.

## 3. Results

### 3.1. Diffusion of Serum

**Figure 1:**
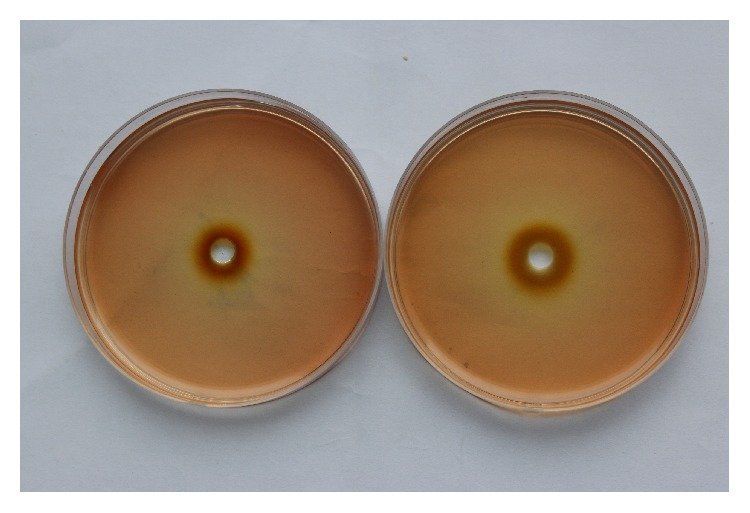
Diffusion of serum in 0.003 mol/L KMnO_4_.

The redox reaction occurred between the antioxidants in serum and the oxidant KMnO_4_. In the potassium permanganate agar, serum diffused from the hole center to all sides. The color of the potassium permanganate agar changed from purple to brown following a decreasing in KMnO_4_. The redox reaction area turned to brown, while the nonredox reaction area turned to red, and the boundary between the two areas was clear.  Figure 1 showed the results of serum diffusion.

### 3.2. Linear Experiment

**Figure 2:**
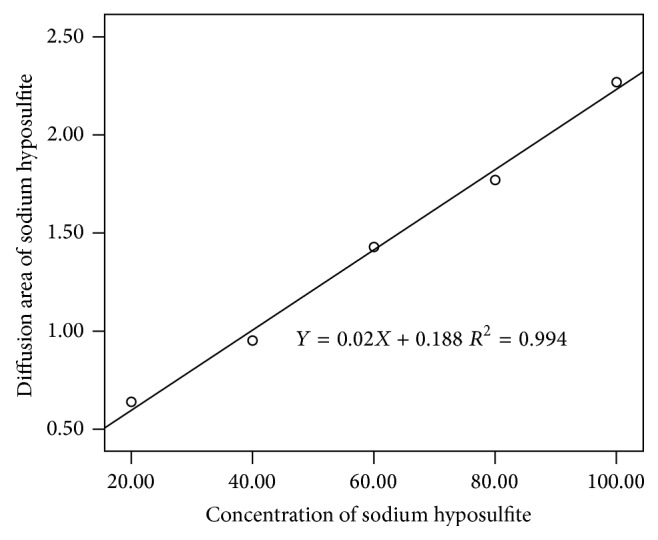
Linear experiment of Na_2_S_2_O_3_ in 0.003 mol/L KMnO_4_.

**Figure 3:**
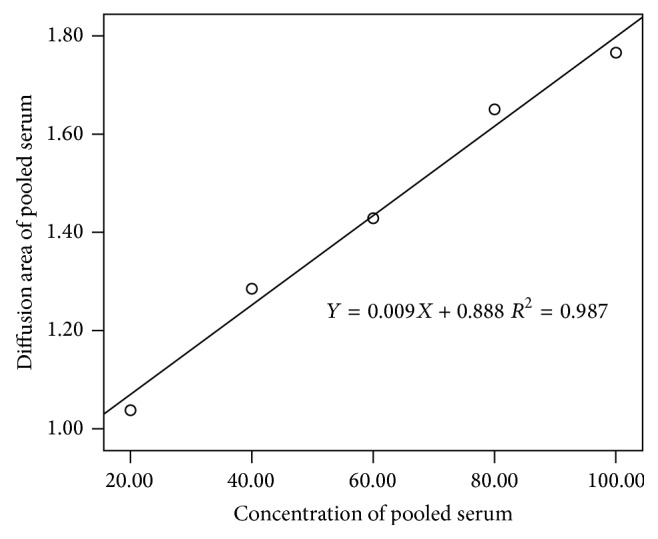
Linear experiment of pooled serum in 0.003 mol/L KMnO_4_.

The accuracy of the potassium permanganate agar method was evaluated by the two linear experiments. Five concentrations of Na_2_S_2_O_3_ diffused in potassium permanganate agar were used to verify the accuracy of the potassium permanganate agar method. Five concentrations of pooled serum diffused in potassium permanganate agar were used to verify the accuracy of the potassium permanganate agar method in matrix substance. The diameter of the brown circle was measured and the area of Na_2_S_2_O_3_ diffusion and serum diffusion was calculated, respectively. In SPSS 13.0, the concentration of Na_2_S_2_O_3_ was set as the *x*-axis and the area of the reaction zone was set as the *y*-axis. A linear relationship between the area of Na_2_S_2_O_3_ diffusion and the concentration of Na_2_S_2_O_3_ was observed (Figure 2). The concentration of pooled serum was set as the *x*-axis and the area of the reaction zone was set as the *y*-axis. A linear relationship between the area of pooled serum diffusion and the concentration of pooled serum was observed (Figure 3). In the linear experiment of Na_2_S_2_O_3_, *R* ^2^ of the equation was 0.994, the slope was 0.02, and the intercept was 0.188. In the linear experiment of serum, *R* ^2^ of the equation was 0.987, the slope was 0.009, and the intercept was 0.888.

### 3.3. Precision Experiment

The area of high level and low level serum diffusion in 0.003 mol/L KMnO_4_ for 20 days was measured. Repeatability (*S*), between-run precision (*S*_rr_), between-day precision (*S*_dd_), and average area ($\bar{x}$) of serum diffusion were measured with the guidance of EP5-A2 document. The coefficient of variation (CV) for *S*, *S*_rr_, *S*_dd_, and $\bar{x}$ calculated (Table 1). The maximum CV of the high level and low level was 6.3% and 5.1%, respectively.

### 3.4. Methodology Comparison

**Figure 4:**
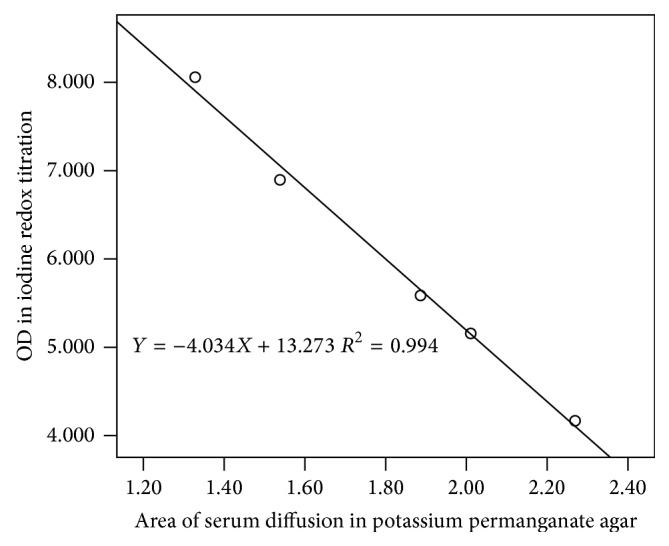
Methodology comparison of the potassium permanganate agar and the iodine redox titration.

In SPSS 13.0, the area of serum diffusion in the potassium permanganate agar was set as the *x*-axis and the OD of iodine redox titration was set as the *y*-axis. There was a good linearity between the two methods. *R* ^2^ of the equation was 0.994, the slope was −4.034, and the intercept was 13.273 (Figure 4).

### 3.5. Results for the ICU Group

Diffusion results in the two groups were shown below (Table 2). In the ICU group, the maximum area of serum diffusion was 2.27 cm^2^, the median (50 percentiles) was 2.01 cm^2^, and the minimum was 0.64 cm^2^. In the healthy group, the maximum area of serum diffusion was 2.27 cm^2^, the median (50 percentile) was 1.77 cm^2^, and the minimum was 1.65 cm^2^. When the results of the two groups were compared, *p* = 0.002 (two tailed).

## 4. Discussion

The redox reaction between the antioxidants in serum and the oxidant KMnO_4_ occurred in this experiment. Serum diffused from the center of the hole to all sides in the potassium permanganate agar. The color of Mn^7+^ is purple and the precipitating MnO_2_ is brown. The color of potassium permanganate agar changed from purple to brown with a decreasing KMnO_4_ and an increasing MnO_2_. The redox reaction area is brown and the nonredox reaction area is red; the boundary between the two areas was clear. The larger the brown area represents more antioxidants were detected in the serum. Different amounts of antioxidants in serum can reflect the amount of TAC in organisms. By measuring the diameter of the brown circle and calculating the area of serum diffusion, it was possible to determine the amount of antioxidants in serum and infer the antioxidant state of the organism.

The linear experiment showed that good linearity was observed. In the linear experiment of the area of Na_2_S_2_O_3_ diffusion versus Na_2_S_2_O_3_ concentration, *R* ^2^ of the equation was 0.994, the slope was 0.02, and the intercept was 0.188. In the linear experiment of the area of pooled serum diffusion versus serum diluted level, *R* ^2^ of the equation was 0.987, the slope was 0.009, and the intercept was 0.888. The linearity of potassium permanganate agar was acceptable and potassium permanganate agar had good accuracy, even in a serum matrix substance. In the precision experiment, the CV of high level within-run was 2.9%, between-run was 6.3%, and between-day was 0 (set estimate of between-run standard deviation to 0 if negative according to EP5-A2 document), while the CV of low level within-run was 3.0%, between-run was 5.1%, and between-day was 0 (set estimate of between-run standard deviation to 0 if negative according to EP5-A2 document). All CV values were less than 10%, and the precision of potassium permanganate agar was acceptable. The diffusion area is dependent on the amount of antioxidants; less amount of antioxidants in sample results in a small diffusion area. In Figure 2, when the Na_2_S_2_O_3_ solution (0.01 mol/L) was diluted to less than 20% of its original concentration, it was difficult to measure the diffusion area and the CV of the results turned larger. Similarly, when serum was diluted to 20%, the antioxidants were also diluted. Trace amount of antioxidants produces small diffusion area, which is hard to measure. To validate our method, the same serum samples at 5 concentration levels were tested with described potassium permanganate agar method compared with previously reported iodine redox titration (Figure 4). Good linearity was observed in OD value from iodine redox titration and diffusion areas from our method. So, good accuracy, precision, and reliability of our described method were proved.

Here, our method was applied in distinguishing serum specimens from ICU patients and healthy subjects. When the area of serum diffusion in the two groups was compared using a nonparametric test, *p* = 0.002 (two tailed) was less than 0.05, showing that the potassium permanganate method was useful for determining total antioxidants in serum. The minimum area of serum diffusion in the healthy group was greater than that in the ICU group, and similar results were observed for the median area of serum diffusion. More antioxidants were detected in the serum of healthy subjects indicating that healthy subjects may have better TAC.

Analysis showed that the potassium permanganate agar method could be better than potassium permanganate titration, as little precipitation of MnO_2_ was generated using this method to judge the end point. Due to the reduced use of reagent and sample in the potassium permanganate agar method, it is possible to apply this method on a large scale in the clinic. Furthermore, the potassium permanganate agar method can exclude interference by external O_2_ due to the covering of liquid paraffin on the surface of the agar. The standard electrode potential of MnO_4_^−^/MnO_2_ was 1.695 V, much higher than 0.77 V for Fe^3+^/Fe^2+^ and 0.16 V for Cu^2+^/Cu^+^, and can detect more antioxidants than the FRAP, especially the CUPRAC assay. The redox reaction in the potassium permanganate agar method occurred in neutral environment instead of acidic environment and can better reflect the state of TAC in organism than FRAP assay. In the potassium permanganate agar method, only KMnO_4_ was required as reagent, and the results were more reliable than those obtained by CUPRAC assay. The results of the CUPRAC assay can be easily influenced by the buffer added to the system. Comparing the potassium permanganate agar method with the TRAP assay and ORAC assay, the procedure in this study is relatively simple, no special devices were required, and measurement of the area of serum diffusion reflected the amount of total antioxidants in serum. Compared with the TEAC assay or DPPH assay, the potassium permanganate agar method had better linearity and precision, indicating that it was a good replacement for these two assays.

## 5. Conclusion

The potassium permanganate agar method had good linearity and precision. The area of serum diffusion shows the concentration of antioxidants and further indicates the serum TAC. By the potassium permanganate agar method, ICU patients and healthy subjects can be distinguished based on the serum diffusion. The potassium permanganate agar method was a new technique for investigating TAC in disease and aging and should be used to determine serum antioxidants in the clinic.

## Figures and Tables

**Table 1:** Average area of serum diffusion, standard deviation of the area of serum diffusion, and coefficient of variation of the area of serum diffusion at different levels (cm^2^).

Sample	$\overset{-}{x}$	SD	CV (%)
High level			
Within-run	1.94	0.06	2.90
Between-run	1.94	0.12	6.30
Between-day	1.94	0.00	—
Low level			
Within-run	1.52	0.05	3.00
Between-run	1.52	0.08	5.10
Between-day	1.52	0.00	—

^*∗*^According to the EP5-A2 document, set *S* ^2^, *S* ^2^_rr_, and *S* ^2^_dd_ to 0 if negative. $\overset{-}{x}$: average area of serum diffusion, SD: standard deviation of the area of serum diffusion, and CV: coefficient of variation of the area of serum diffusion.

**Table 2:** Diffusion results in the healthy group and ICU group (cm^2^).

Group	Min	Percentiles			Max	*p*
		25	50	75		
Healthy	1.65	1.89	2.01	2.11	2.27	0.002
ICU	0.64	1.65	1.77	2.01	2.27	

^*∗*^Min: the minimum area of serum diffusion, Max: the maximum area of serum diffusion.
